# Supplementary material for: Developing Recommendations for Cumulative Endpoints and Lifetime Use for Research Animals
Source: Animals (Basel). 2021 Jul 7;11(7):2031. doi: 10.3390/ani11072031 (PMC8300189; doi:10.3390/ani11072031)
Supplement: Supplementary file 1 [file animals-11-02031-s001.zip › animals-1234798-supplementary.pdf]

Supplemental Table S1. The lifetime use and cumulative endpoints for research and teaching animals survey.

Part	Question
Part A: Demographics of Respondents	1. Please select the region that you live and work from the list.
	2. Please select the range below that includes your age.
	3. Please indicate your gender.
	4. Please indicate the type of institution that you work in.
	5. Please indicate your primary job function (e.g., veterinarian, technical supervisor, researcher, etc.)
	6. A. Do you use animals in your primary job function?<br>7. If yes to 6A., please indicate that species that you work with from the list.
Part B: Current Policies or Procedures for Experimental Endpoint Decision Making	8. Does your animal ethics committee have a formal experimental endpoint policy for research animals held at your facility?
	9. Please indicate which species are covered by the endpoint policy from the list.
	10. Please indicate which of the following considerations may be used to trigger an endpoint discussion for an animal whether on a holding, breeding, teaching, or research protocol.
	11. Other than euthanasia, what types of options have been instituted at your facility when animals reach a pre-determined study endpoint?
Part C: Lifetime Use of Animals in Research and Teaching<br><br>Please review the following statements and select the answer that best approximates your opinion (Strongly agree, Agree, Neither Agree nor Disagree, Disagree, Strongly Disagree).	12. Animals of any species should not be removed from the research program, regardless of use, as this necessitates purchasing new replacement animals (i.e., contradicts reduction principles).
	13. Provided that there is no physical trauma, such as rectal mucosal tears, it is acceptable for students to perform rectal examinations on teaching mares twice weekly for the mare's entire institutional life (e.g., 10+ years).
	14. As long as the maximum blood volume guidelines are adhered to, it is acceptable to perform an unrestricted number of venipunctures on a rat in a pharmacokinetic study.
Part D: Quality of Life and Lifetime Use Endpoints	15. Please select from the following list all methods that apply to your facility as they pertain to quality of life or lifetime use endpoints.

	a. My institutional animal ethics committee has a guidance document for determining animal disposition at study end that includes the use of objective metrics, such as number of studies conducted with animal, number of needle sticks, number of study days, protocol invasiveness, total blood volume provided over a lifetime, etc.<br>b. My institutional animal ethics committee has established adoption and/or retirement criteria for a species, regardless of study use (e.g., based on animal age, time in facility, etc.).<br>c. My facility has an assessment protocol for scoring animals exhibiting aversive behaviors to procedures (e.g., vocalization during blood collection, excessive trembling, struggling).<br>d. My institutional animal ethics committee periodically discusses quality of life of aging and long-term housed research and teaching animals.<br>e. Animal care and veterinary personnel at my facility regularly review quality of life and endpoint decision-making for aging and long-term housed research and teaching animals.
Part E: Reduction versus Refinement	16. In a long-term study requiring regular blood collection requiring 100+ collections without the use of a catheter or vascular access port, which would be the most appropriate use of animals?<br>17. In a long-term study with liver biopsies obtained via percutaneous ultrasound-guided biopsy under general anesthesia, resulting in 10+ biopsies, which would be the most appropriate use of animals?<br>18. In a colony of animals kept for repeated, minimally invasive studies, in which the animals are used for multiple sample collections, which consideration for animal use is most appropriate?<br>19. If animals need to be single housed for 1-2 weeks during the course of these studies, does this change your answer?
Part F: Conclusions	20. Would you support the adoption of species-specific lifetime use guidelines at your institution?<br>21. Do you have any other comments to make regarding experimental endpoints and

	considerations for cumulative lifetime use of research animals?
